# Surface Plasmon Coupling in GaN:Eu Light Emitters with Metal-Nitrides

**DOI:** 10.1038/s41598-018-31821-8

**Published:** 2018-09-06

**Authors:** Ioannis E. Fragkos, Nelson Tansu

**Affiliations:** Center for Photonics and Nanoelectronics, Department of Electrical and Computer Engineering, Lehigh University, Bethlehem, PA 18015 USA

## Abstract

Metal-nitrides of hafnium nitride (HfN), zirconium nitride (ZrN) and titanium nitride (TiN) are investigated as plasmonic materials to enhance the internal quantum efficiency of a GaN:Eu red light emitter. Theoretical calculations are performed to evaluate the surface plasmon polariton dispersion relation and Purcell enhancement factor for a single metal-nitride layer on top of the GaN:Eu emitter. Our findings suggest that among the metal-nitrides investigated in this study, TiN is the most promising candidate for use as plasmonic material to increase the internal quantum efficiency in GaN:Eu red light emitters.

## Introduction

Europium-doped gallium nitride (GaN:Eu) based light emitters have drawn much attention in the field of photonics due to the sharp luminescence of Eu^+3^ ions emitting in the red spectral regime^1–8^.The recent progress in the GaN:Eu based material has resulted in promising direction for its implementation as a candidate for active region in red light-emitting devices (LEDs)^9–17^. However, regardless the evolution and the improvements of the GaN:Eu devices over the years, the progress in the electrically-driven devices has been limited to low peak internal quantum efficiency (*η*_*IQE*_) and drooping behavior of external quantum efficiency (*η*_*EQE*_) with increasing current density^17^.

In our recent work^18^, we developed a current injection efficiency model (*η*_*injection*_) to understand the governing parameters affecting the internal quantum efficiency and efficiency-droop characteristics in the electrically-driven GaN:Eu LEDs. This model^18^ also allows one to reveal the fundamental distinct factors that affect the efficiency characteristics in the optically-pumped and electrically-driven GaN:Eu devices. Through this model^18^, we identify that the saturation of the excited Eu^+3^ ions in the GaN host - resulted either by optical excitation or electrical injection in the GaN host - as one of the key factors limiting the peak internal quantum efficiency and droop issue at high injection current level. These limiting factors^18^ resulted the low current injection efficiency in electrically-driven GaN:Eu LED, which in turn led to a significant reduction in both its internal and external quantum efficiencies.

In addition, we proposed experimental methods on how to increase the external quantum efficiency of these devices. Our studies^18^ pointed out the reduction in the radiative life time of Eu^+3^ ions (*τ*_*rad*_) reduces the saturation issue of the Eu^+3^ ions up to higher current density; such approach enables the ability to achieve high current injection level and minimize the drooping issue at higher current density (for electrically-driven GaN:Eu devices) and at higher photon flux (for optically-excited devices). The reduction in the *τ*_*rad*_ also increases the radiative efficiency (*η*_*radiative*_) of the Eu^+3^ ions, which in turn results in a further enhancement of the internal quantum efficiency (*η*_*IQE*_ = *η*_*injection*_ ∙ *η*_*radiative*_) of the device.

The reduction of the radiative lifetime of Eu^+3^ ions can be physically achieved by using the coupling of the emitted electromagnetic (EM) mode to a surface plasmon (SP) of a conductive layer, spaced at a distance from the emitter, thus creating a surface plasmon polariton (SPP). Through this coupling, the radiative efficiency of the system is enhanced due to increase of photon density states near the surface plasmon energy *E*_*sp*_ - a phenomenon known as the Purcell effect^19^. It is important to notice that the coupling mechanism of the emitter to the surface plasmons of the conductive layer, usually requires special configurations in order to achieve phase matching conditions of the emitted photon of the light source and the surface plasmon of the conductive layer. However, it has been shown that if the light source is close to the conductive layer within the wavelength scale, a SPP can be generated via direct energy transfer from the light source to the surface plasmon. Therefore, for the case where the conductive layer is placed on top of a light source in a relatively short distance from it, no special configurations are required to generate SPPs^20–22^.

The concept of SP coupling to the active materials in III-Nitride semiconductors resulting in an enhanced radiative efficiency has been reported^23–29^. More specifically, metallic thin layers such as silver (Ag) and gold (Au) have been deposited on top of InGaN multiple quantum wells for increasing the efficiency of the emitter in the ultraviolet (UV) and visible spectral regimes^23–29^. However, despite the popularity of these noble metals as the metallic plasmonic material choices for applications in the UV and visible spectral regime, such materials are unsuitable for plasmonic applications in the red and near infrared spectral regime attributed to high Ohmic losses^30,31^. In contrast, the transition-metal nitrides, such as titanium nitride (TiN), hafnium nitride (HfN) and zirconium nitride (ZrN), are promising candidates as low-loss plasmonic materials in the visible and near-IR spectral regimes attributed to the ability for achieving negative real permittivity values at relatively lower carrier concentrations^32–37^. In addition, these materials offer a wide tunability of their dielectric properties, usually through the variation of the deposition parameters^32–37^.

In this work, we theoretically investigate the use of metal nitrides - TiN, HfN and ZrN - as plasmonic materials in the red spectral regime. More specifically, we study the effect of the coupling of the surface plasmons of the metal-nitride to the GaN:Eu based red light emitter and its impact on the internal quantum efficiency of this particular type of red light emitter. A comparison among the metal nitrides is made, and is found that TiN is the most suitable selection for applications in the characteristic photon energy of the GaN:Eu emitter at ~ 2 eV. Consequently, the study is proceeded to investigate the effect of the TiN layer thickness (d_TiN_) and GaN spacer thickness (d_spr_) on the SPP dispersion relation and Purcell factor. The impact on the electrically-driven device characteristics for TiN-based surface plasmon coupled on GaN:Eu LEDs is also presented.

## Simulation Method and Results

**Figure 1:**
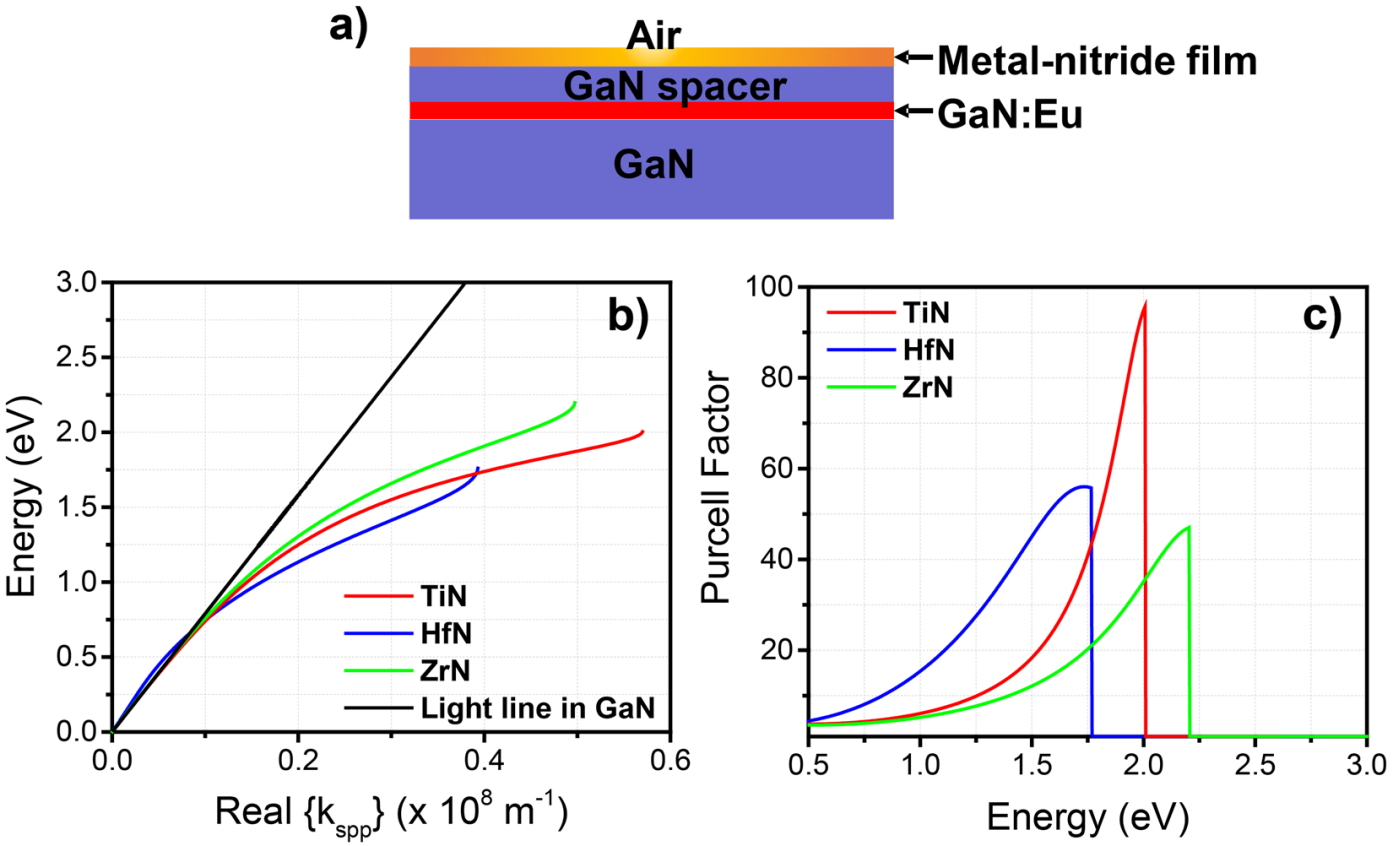
(**a**) Schematic of the structure used for the simulations. (**b**) Energy dispersion relation of the surface plasmon polariton (SPP) for different metal-nitrides. The thickness of the metal-nitride film was set at 20 nm while the GaN spacer thickness of was set at15 nm. (**c**) Purcell factor for different metal-nitrides films on top of the GaN:Eu red light emitter.

For our theoretical investigation we consider the structure depicted in Fig. 1(a). The metal-nitride layer is placed on top of a GaN/GaN:Eu/GaN emitter, where the last layer of GaN serves as the spacer between the GaN:Eu and metal-nitride regions. The SPP dispersion relation is computed by solving the Maxwell equations in the GaN/GaN:Eu/GaN active layer, metal-nitride plasmonic layer, and air region with the appropriate boundary conditions. After solving the SPP dispersion relation, the electric field profile is evaluated to calculate the Purcell enhancement factor; this method is similar to work presented in ref.^23^. The simulations are performed assuming the dielectric functions of GaN:Eu and GaN regions as identical and equal to *ε*_*rGaN*_ = n^2^_*GaN*_, where *n*_*GaN*_ = 2.5 is the refractive index of GaN. For the dielectric function of the metal-nitrides, a Drude-Lorentz model is used according to the following formula:

$${\varepsilon }_{rMN}(\omega )={\varepsilon }_{{\rm{\infty }}}-\frac{{{\omega }_{p}}^{2}}{{\omega }^{2}-i\omega {{\rm{\Gamma }}}_{p}}+\sum _{j=1}^{m}\frac{{f}_{j}{{\omega }_{j}}^{2}}{{{\omega }_{j}}^{2}-{\omega }^{2}+i\omega {{\rm{\Gamma }}}_{j}}$$

where the *ε*_*∞*_ is the background constant permittivity at high frequency, the *ω*_*p*_ is the plasma frequency of the metal-nitride, the *Γ*_*p*_ is the damping factor, and the *ω*_*j*_ is the frequency of the Lorentz oscillators with strength *f*_*j*_ and damping factor *Γ*_*j*_. The parameters of the dielectric function of the individual metal-nitride materials investigated in this study are shownin Table 1.

**Table 1:** Parameters of the Drude-Lorentz model for the different metal-nitrides investigated in this study.

Parameter	HfN	TiN	ZrN
ε_∞_	2.5	4.855	3.465
*ω*_*p*_ [eV]	5.71	7.9308	8.018
*Γ*_*p*_ [eV]	0.6878	0.1795	0.5192
*ω*_1_ [eV]	4.60	4.2196	5.48
*f*_1_	1.20	3.2907	2.4509
*Γ*_1_ [eV]	2.65	2.0341	1.7369
Reference	^37^	^36^	^36^

Prior to the theoretical investigation it is important to mention that the structure studied here (Fig. 1 (a)) is basically the active region of a GaN:Eu based device. In addition, in our study we have excluded any possible cavity effects on the Purcell enhancement factor. The structure depicted in Fig. 1(a) does not contain any highly reflective layers (i.e reflective metals, distributed Bragg reflectors), as well as does not meet the criteria for the lowest resonant cavity frequency: the required cavity width for the lowest resonant frequency is λ/2 (where the λ is the characteristic emitted wavelength of the light source), while the total width of the structure depicted in Fig. 1(a) is much less than the above required condition.

Figure 1(b) depicts the effect of different metal-nitride materials on the SPP dispersion relation. The thickness of the metal-nitride layer was fixed at 20 nm while the thickness of the GaN spacer was fixed at 15 nm. It can be seen that the SPP dispersion relation approaches an asymptotic limit which corresponds to the characteristic surface plasmon polariton energy (*E*_*sp*_). Among the metal-nitrides the energy of the SPP of the TiN is very close to the characteristic photon energy of the GaN:Eu emitter (~2 eV) in the red spectral regime. In contrast the ZrN and HfN present a characteristic *E*_*sp*_ which is in the green (~2.2 eV) and deep red (~1.70 eV) spectral regime respectively. In addition, as it is shown in Fig. 1(c), TiN presents high Purcell factor at the characteristic energy of *E*_*sp*_ as compared to the other metal-nitrides. It is important to mention that this comparison of metal-nitrides aims on the selection of the appropriate material for plasmonic application only in the spectral regime of the characteristic photon energy of GaN:Eu emitter at ~2 eV. Therefore, TiN is found to be a suitable plasmonic material for the surface plasmon coupling with the GaN:Eu based red light emitter. The use of the other metal-nitrides presented in this study can be used for similar applications in the spectral regime of their characteristic asymptotic limit of *E*_*sp*_.

**Figure 2:**
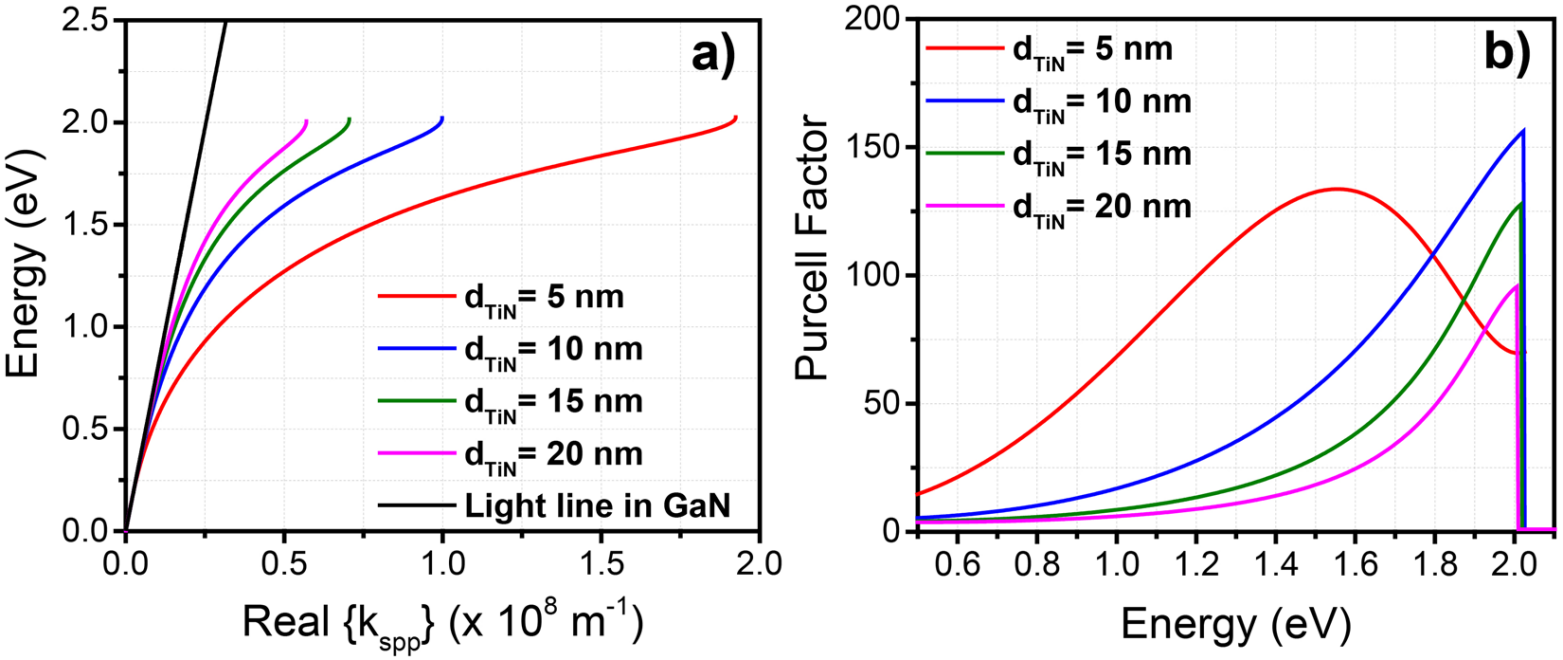
(**a**) Energy dispersion relation of the surface plasmon polariton (SPP) for different TiN thickness (d_TiN_) with GaN spacer thickness of d_spr_ = 15 nm. (**b**) Purcell factor for different TiN thickness (d_TiN_) with GaN spacer thickness of d_spr_ = 15 nm.

Figure 2(a) depicts the effect of different TiN layer thickness on the SPP dispersion relation for the case of *d*_*spr*_ = 15 nm. By decreasing the TiN thickness, the SPP curve is pushed down at lower energies while maintaining the asymptotic limit at *E*_*sp*_ ~ 2.0 eV. In general, for a thin conductive layer surrounded by dielectrics, collective plasma oscillations localized at the metal/dielectric and metal/air interfaces exist. The thinner the conductive layer is, the stronger the coupling between the SPPs at the two interfaces becomes resulting in a larger energy separation of the two SPPs^27^. As the energy approaches the *E*_*sp*_, the penetration depth of the electric field of the SPP in the conductive layer significantly reduces resulting in the non-interaction of the two SPPs at the two interfaces. Hence, the limit of *E*_*sp*_ is independent of the conductive layer thickness.

Figure 2(b) depicts the Purcell factor for various TiN thicknesses for the case of *d*_*spr*_ = 15 nm. As the energy of the SPP increases towards *E*_*sp*_, the Purcell factor increases due to the increased surface plasmon density of states (SPDS). The maximum value of the Purcell factor is obtained at the limit of *E*_*sp*_, where the SPDS has its maximum value. Note that the SPDS is proportional to (*dE*/*dk*)^−1^ of the dispersion curve shown in Fig. 2(a). At energies above *E*_*sp*_, the GaN/TiN interface cannot support a guided SPP mode, hence the Purcell factor drops to unity. In addition, by decreasing the TiN thickness, the Purcell factor can be obtained for values higher than 100. In contrast, by reducing the TiN thickness beyond 10 nm, the Purcell factor drops at energy ~*E*_*sp*_, while it becomes broader at lower energies. This behavior is attributed to the lower energies of the SPP for the case of *d*_*TiN*_ = 5 nm as compared to those with *d*_*TiN*_ > 5 nm, as shown in Fig. 2(a).

As shown in Fig. 2(b), the use of thinner TiN layer results in larger SPP wavevector (*k*_*spp*_) with high Purcell factor; however, such condition comes at the expense of the SPP propagation length (*L*_*spp*_). The propagation length *L*_*spp*_ of ~4.05 nm is obtained for the case of *d*_*TiN*_ = 5 nm. As the *d*_*TiN*_ is increased to 15 nm (or 20 nm), the *L*_*spp*_ increases to ~11.7 nm (~15 nm). Despite the relatively low propagation length, the out-coupling of the SPP into the air can be achieved through scattering via the roughness of the TiN/GaN interface^25,38^.

**Figure 3:**
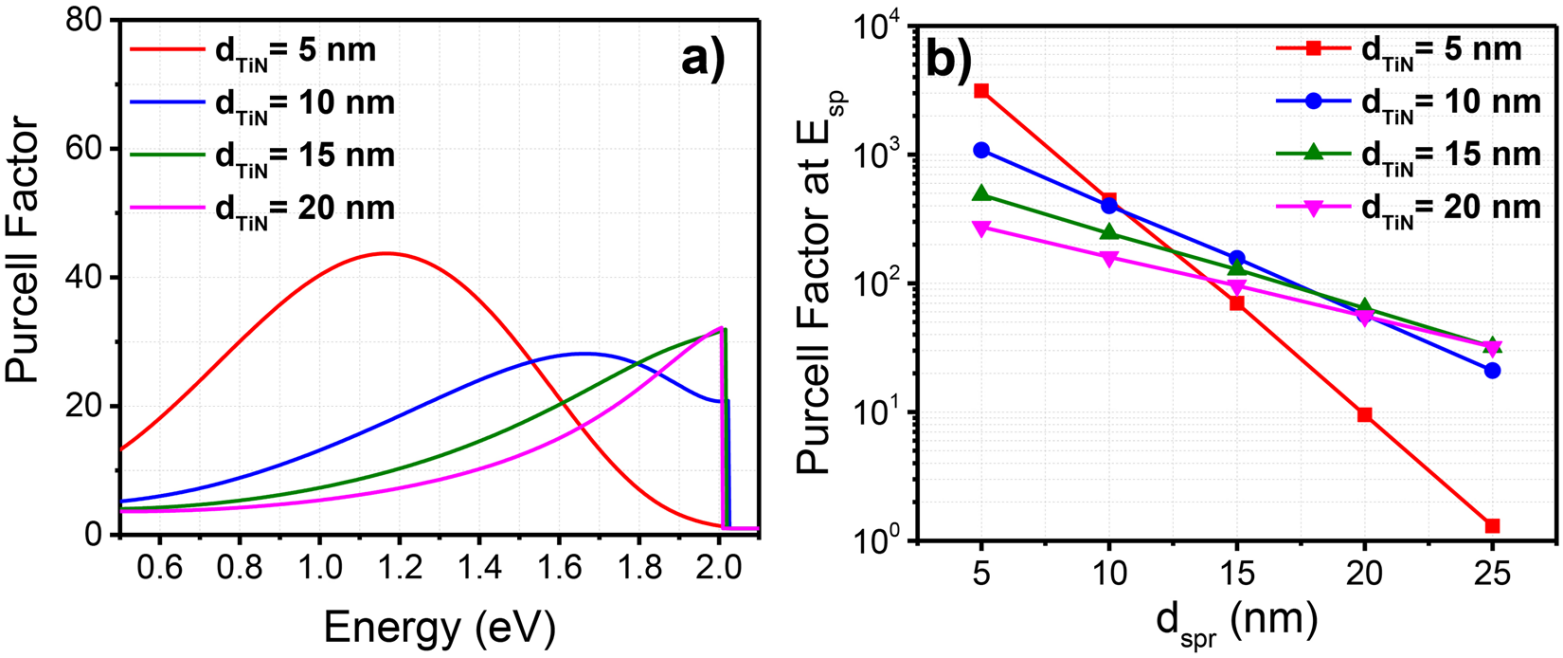
(**a**) Purcell factor for different TiN thickness (d_TiN_) with GaN spacer thickness of d_spr_ = 25 nm. (**b**) Purcell factors at the asymptotic limit of E_sp_ versus different GaN spacer thickness (d_spr_) plotted for different thickness of TiN (d_TiN_).

In Fig. 3(a), a similar dependency of the Purcell factor on the TiN thickness is observed for a different spacer thickness of *d*_*spr*_ = 25 nm. Figure 3(b) depicts the Purcell factors at the asymptotic limit of *E*_*sp*_ versus different spacer thickness, plotted for different TiN thicknesses. A reduction of the Purcell factor with the spacer thickness is observed for all cases. In general, a thicker spacer corresponds to a larger separation of the TiN and GaN:Eu layers, which in turn results in a weaker coupling of the GaN:Eu region to the SP layer. The interplay role of the conductive and the spacer layer thicknesses has also been demonstrated for the case of GaN/Ag/air interfaces^24^.

The dielectric properties of TiN strongly depend on the deposition conditions^39–45^. By tuning the deposition conditions of TiN on top of GaN, the desirable dielectric properties of TiN can be achieved. In this way the surface plasmon frequency can be designed to cover a wide range of the visible spectral regime. In contrast to the single TiN layer presented in this work, two different layers of TiN with different dielectric functions can be used to tune the asymptotic limit *E*_*sp*_. The concept of a double layer (DL) has been demonstrated for the case of the InGaN QWs where the thickness of the individual layers of the DL varied to tune the surface plasmon energy *E*_*sp*_^27,28^. A similar concept can be applied for the case of a double TiN layer on top of GaN based light emitters, including GaN:Eu and InGaN QWs, which could potentially increase the Purcell factor over a wide range in the visible spectral regime.

## Impact of Purcell Factor on Internal Quantum Efficiency

**Figure 4:**
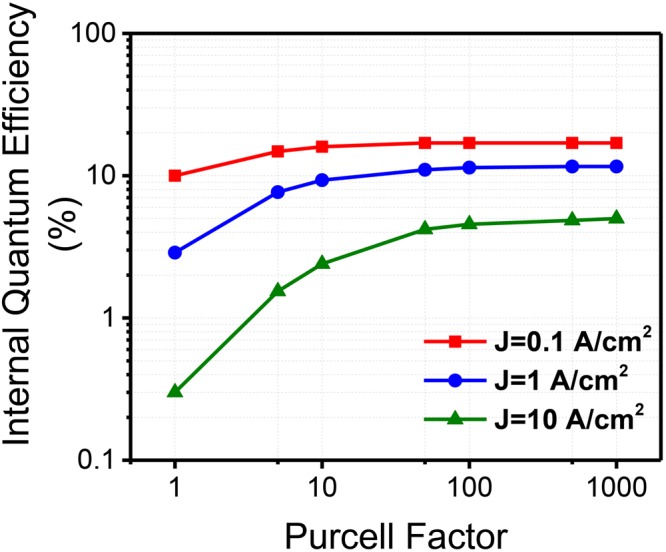
Internal quantum efficiencies of electrically-driven GaN:Eu LED taking into consideration current injection efficiency as a function on Purcell enhancement factor plotted for three different current densities (J).

The analysis of the Purcell factor in the internal quantum efficiency of the electrically-driven GaN:Eu LEDs is presented in Fig. 4, in order to quantify the improvement presented from the use of surface plasmon structure. The internal quantum efficiency of a GaN/GaN:Eu/GaN device was calculated in a similar approach to ref.^46^. The introduction of surface plasmon coupled active region in GaN:Eu results in an order of magnitude increase in the internal quantum efficiency of the electrically-driven devices, and provides a reduction of efficiency-droop up to relatively high current density (*J*).

A large surface plasmon coupled structure (Purcell factor ~1000) results in peak internal quantum efficiency ~20% for *J* = 0.1 A/cm^2^. The droop suppression from the Purcell effect is found to be more significant at higher current densities. Specifically, a Purcell factor of 1000 results in an increase of the internal quantum efficiency by ~16 times at a current density of *J* = 10 A/cm^2^, while the same Purcell factor results in only ~1.7 times higher improvement for *J* = 0.1 A/cm^2^. For the structure without any surface plasmon coupling (reference case, with Purcell factor = 1), a significant reduction (~30 times) in the internal quantum efficiency is observed as the current density increases from *J* = 0.1 A/cm^2^ to *J* = 10 A/cm^2^. In contrast, the reduction of only ~3.5 times in the internal quantum efficiency was observed in the structure having a large Purcell factor (~1000) for the same current density range (*J* = 0.1 A/cm^2^ to *J* = 10 A/cm^2^). As mentioned earlier, the changes in the Purcell factor correspond in changes of the radiative lifetime of the GaN:Eu region (i.e Eu^+3^ ions) which affect the radiative efficiency of the system. In addition, a change in the current injection efficiency will also occur resulting in an overall change of the internal quantum efficiency of the GaN:Eu device.

In addition to the need to achieve high internal quantum efficiency in the electrically-injected surface-plasmon GaN:Eu LED, numerous approaches for achieving high extraction efficiency needs to be considered. The metal-nitrides and other surface plasmon metals have surface roughness of ~ 1 nm via evaporation or sputtering^25,28,35,38^, and such roughness contributes greatly to the light scattering into and out of the surface plasmon layer. It is expected that the use of thin-film flip-chip (TFFC) LEDs can be implemented in surface-plasmon structure for enabling extraction in the order of ~ 75% or higher^47–49^. The advantage presented in this work by using surface plasmon coupling to increase the IQE in the electrically-injected GaN:Eu LED can be incorporated in the TFFC configuration.

**Figure 5:**
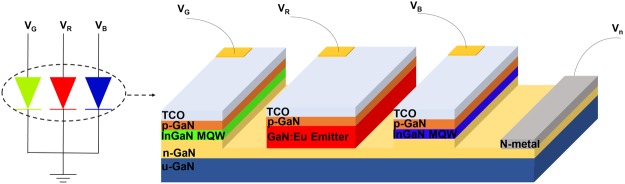
Concept of monolithically integrated white LED based on GaN material. The high efficiency blue and green InGaN QW can be monolithically integrated with the high efficiency red GaN:Eu emitter.

The development for high efficiency red light emitters based on GaN:Eu or In_x_Ga_1−x_N QW active region, is mandatory for the monolithic realization of GaN-based white LEDs. The solutions for challenges to achieve InGaN-based red emitters are important and still being pursued. However, the use of GaN:Eu LEDs may provide an interesting advantage over InGaN, namely: i) narrower linewidth red spectral emission, and ii) less temperature-sensitivity to the emission wavelength^10–13,17,50,51^. The availability of red emitters based on GaN:Eu LEDs provides a pathway for integration with the more established InGaN-based blue and green emitters. Figure 5 shows the integration method that can be pursued for GaN-based display emitters with integration of InGaN-based LEDs and red-emitters based on GaN:Eu materials. Such integration can provide a solution for individually-addressed emitter in the three colors grown by selective-area epitaxy.

## Conclusions

The metal-nitrides of hafnium-nitride (HfN), zirconium nitride (ZrN) and titanium nitride (TiN) have been investigated as plasmonic materials to enhance the internal quantum efficiency of the GaN:Eu red light emitter. It was found that among those metal-nitrides, the TiN is the most promising material candidate for surface plasmon coupling to the GaN:Eu red light emitter. Through the tuning of the TiN and GaN spacer thickness, Purcell factors as high as 1000 can be achieved at a photon energy ~*E*_*sp*_. The coupling of the active region of a GaN:Eu LED to the surface plasmon of the TiN layer is expected to result in significant increase in the internal quantum efficiency of the electrically-driven devices. This approach will provide a pathway for achieving 20% internal quantum efficiency in electrically-driven devices. A significant reduction in drooping at high current density is expected in the surface-plasmon coupled GaN:Eu electrically-driven LED. The droop suppression in the electrically-driven surface plasmon coupled device is expected to improve by ~16 times over that of the reference devices without employing surface plasmon coupling.
